# Accounting for social and environmental drivers of neurodevelopment: lessons from a pilot study in a pediatric Colombian cohort

**DOI:** 10.1038/s41390-025-04452-9

**Published:** 2025-11-04

**Authors:** Regan Andringa-Seed, Laura Calderon Suarez, Elizabeth Corn, Heather Gordish-Dressman, Meagan E. Williams, Pablo Reyes, Colleen Peyton, Madison M. Berl, Margarita Arroyave-Wessel, Michael E. Msall, Carlos Cure, Sarah B. Mulkey

**Affiliations:** 1 Zickler Family Prenatal Pediatrics Institute, Children’s National Hospital, Washington, DC USA; 2 Division of Biostatistics and Study Methodology, Children’s National Hospital, Washington, DC USA; 3 Department of Psychiatry and Mental Health, Hospital Universitario San Ignacio and Pontificia Universidad Javeriana Faculty of Medicine, Bogotá, Cundinamarca Colombia; 4 Department of Physical Therapy and Human Movement Sciences, Northwestern University, Chicago, IL USA; 5 Division of Pediatric Neuropsychology, Children’s National Hospital, Washington, DC USA; 6 Department of Psychiatry and Behavioral Sciences, The George Washington University School of Medicine and Health Sciences, Washington, DC USA; 7 Kennedy Research Center on Intellectual and Neurodevelopmental Disabilities, University of Chicago Comer Children’s Hospital, Chicago, IL USA; 8 BIOMELab SAS, Barranquilla, Atlántico Colombia; 9 Department of Neurology, The George Washington University School of Medicine and Health Sciences, Washington, DC USA; 10 Department of Pediatrics, The George Washington University School of Medicine and Health Sciences, Washington, DC USA

## Abstract

**Background:**

Child neurodevelopment is shaped by complex social, environmental, and biological factors. Few studies have characterized these factors in Latin American populations. We aimed to evaluate the impact of social and environmental factors on neurodevelopmental outcomes in 5–6-year-old children from three geographic locations in Colombia.

**Methods:**

Our pilot, cross-sectional study included 105 typically developing Colombian children from two urban cities and one rural municipality. Measures assessed domains of IQ (Wechsler Preschool and Primary Scale of Intelligence—WPPSI), executive functioning, psychosocial functioning, and a range of home environmental factors. We compared outcomes across locations and the impact of covariates using linear regression models.

**Results:**

Including covariates, adjusted mean WPPSI IQ was significantly lower in children living in a rural town (80.41 ± 2.07) compared to either city (96.30 ± 2.76; 91.69 ± 2.90). Maternal education, location, dietary diversity, and home resources explained 46% of the variance in IQ. Outcomes in other domains were significantly different, but functioning was all within age expectations across groups.

**Conclusion:**

Significant disparities in cognitive and other outcomes exist among typically developing Colombian children, influenced by location, maternal education, and home resources. Future research should ensure well-matched control populations and use culturally appropriate, validated assessments to minimize social, cultural, and environmental confounding.

**Impact:**

- Maternal education, dietary diversity, total home resources, and location account for approximately 50% of the neurodevelopmental variability of children from urban and rural Colombian cohorts.
- Typically developing children from a rural community in Colombia have a lower Full-Scale IQ than peers from urban cities, even after including multiple covariates.
- Future research should continue to study the multiple factors that impact neurodevelopment, particularly in low- and middle-income countries.
- Well-matched control populations and the use of culturally appropriate, validated assessments are important to minimize confounding factors in neurodevelopmental outcomes research, as statistical correction is not sufficient to fully account for these differences.

## Introduction

Home environment and community factors are recognized as crucial drivers of neurodevelopment across the lifespan. Studies show that neighborhood disadvantage, food insecurity and malnutrition, adverse childhood experiences, and lack of caregiver engagement, among numerous other factors, are associated with lower cognitive performance, lower motor skills, lower educational attainment, and an increased risk for internalizing and externalizing disorders.^1–5^ Given the strong influence of these contextual characteristics, it is important to account for them in longitudinal neurodevelopmental outcome studies.

One common method of accounting for these factors, particularly in studies with heterogeneous participant populations, is to include sociodemographic and environmental characteristics as covariates in analysis models.^6^ However, there is limited standardization in practices of adjusting for social environmental characteristics, which limits the generalizability of conclusions and comparability between studies.^7^ Particularly in global health studies, this practice may not be adequate, as the subjective decision of which factors to include, and how to measure or categorize them, can introduce significant bias into research findings.^8^

**Fig. 1:**
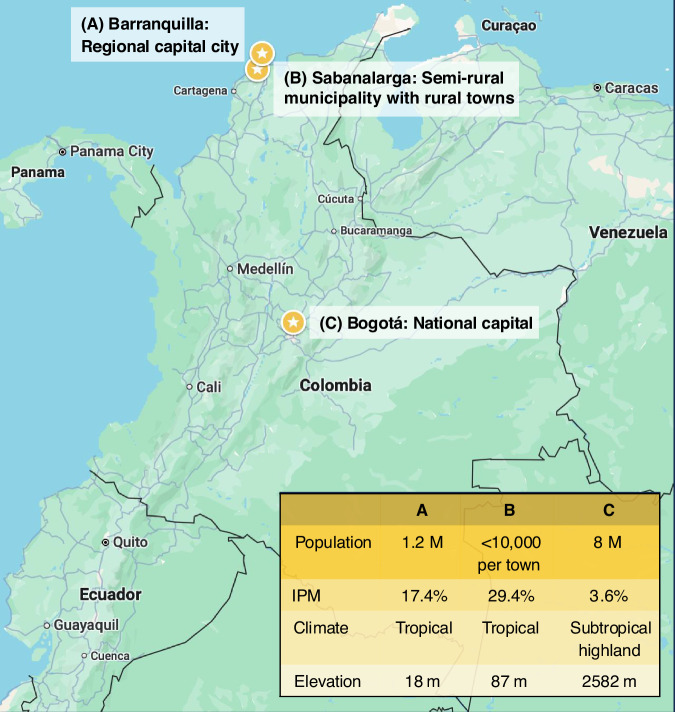
Locations in Colombia from which cohorts of typically developing children were recruited. IPM Índice de Pobreza Multidimensional (Multidimensional Poverty Index). The IPM is a composite measure that assesses poverty beyond income, including indicators related to education, health, living conditions, and access to basic services.^28^ A higher IPM score indicates greater levels of multidimensional poverty. Map of Colombia and neighboring countries sourced from maps.google.com.

In this study, we present the neurodevelopmental outcomes of three groups of typically developing children from three different locations in Colombia—Bogotá, Barranquilla, and Sabanalarga (Fig. 1)—to explore how location and other factors contribute to child neurodevelopment. Bogotá is the capital of the country, a large urban center with a highly concentrated population and relatively greater access to infrastructure, education, and healthcare.^9,10^ Barranquilla, located on the Caribbean coast, is the capital of the department of Atlántico and is a growing city known for its cultural significance and economic activity.^10,11^ Sabanalarga, in contrast, is a smaller, semi-rural municipality in the Department of Atlántico where access to services, transportation, and other resources may be more limited.^12^

We focused on children aged 5–6 years to capture an important stage of development as they begin formal education. This age marks a key milestone in cognitive, emotional, and behavioral maturation, while also reflecting the cumulative impact of early-life experiences, including health, nutrition, home environment, and caregiving practices.^13^ Importantly, children at this age typically demonstrate sufficient attention and cooperation to complete more comprehensive and reliable neuropsychological assessments.^14–16^ Moreover, assessing developmental status at this age is crucial for identifying children who may benefit from early interventions prior to or during the early stages of schooling, which can have long-term implications for academic achievement and social integration.^13^ In Colombia, early childhood health services include standardized newborn screening and routine developmental surveillance integrated into the national primary healthcare system. As outlined in the 2024 Resolution No. 207, newborn screening covers congenital endocrine and metabolic disorders, hearing loss, visual impairments, and congenital heart disease.^17^ In addition, pediatric well-child visits (Controles de Crecimiento y Desarrollo) are scheduled regularly from birth through early childhood. These visits include assessments of motor, language, cognitive, and socioemotional milestones, and are conducted by healthcare professionals to detect early signs of developmental delays.^18^ However, the reach of these services varies considerably across regions, particularly in rural and underserved areas, potentially leading to undetected developmental concerns in vulnerable children.^19,20^

Neurodevelopmental disorders (NDDs) such as autism spectrum disorder (ASD), attention deficit hyperactivity disorder (ADHD), and developmental delays are prevalent in early childhood, and an estimated 95% of children affected by NDDs live in low- and middle-income countries (LMICs).^21^ A recent meta-analysis estimated that 18.8% of children under 5 in LMICs experience developmental delays.^22^ In Colombia, national prevalence estimates remain limited. According to the Ministry of Health’s current clinical protocol, approximately 16% of children under 15 are estimated to have some form of developmental disorder.^23^ A 2024 national health registry analysis reported an overall ASD prevalence of 13.8 per 10,000 children aged 4–14 in Colombia; lower rates were reported in rural areas of the country than urban areas, which suggests disparities in access to ASD diagnosis.^24^ Similarly, rates of ADHD vary across studies and regions and are reported at a range of 3.4–20% of Colombian children.^25^

Like many countries, the distribution of wealth and resources in Colombia is not equitable across all cities and regions.^26,27^ Capital cities like Bogotá and Barranquilla tend to have more robust healthcare and educational systems and a higher concentration of opportunities for their residents. In contrast, more rural regions like Sabanalarga face challenges such as lower educational attainment, higher rates of poverty, and less access to specialized health services.^26,27^ This disparity is reflected in the Multidimensional Poverty Index (IPM, Índice de Pobreza Multidimensional^28^), as shown in Fig. 1. We anticipate that neurodevelopmental outcomes differ among these culturally and socioeconomically diverse locations; however, multi-domain child developmental outcomes have not been well-characterized in these regions.

Using well-characterized cohorts, we compare neurodevelopmental outcomes among children in three locations, accounting for the complex social and environmental factors that differentiate them. We hypothesized that neurodevelopmental outcomes would differ among groups, with lower scores in the rural cohort from Sabanalarga than those from Bogotá and Barranquilla. We anticipated that all covariates included in this analysis (parental education, nutrition, physical and time-based home resources, breastfeeding, and location) would contribute to the observed variability between groups across cognitive, social-emotional, and executive functioning domains.

## Methods

### Study population

We performed a cross-sectional study of 105 healthy, typically developing Colombian children from three distinct regions of the country. Twenty-six children were recruited from a private school in Bogotá, the national capital of Colombia in a mountainous region of the country with a population of approximately 8 million (Fig. 1). Twenty-four children were recruited from a public charter school in Barranquilla, a smaller capital city (population 1.2 million) in the coastal Department of Atlántico (Fig. 1). Finally, 55 children were recruited from public schools in the semi-rural and rural towns of La Peña, Aguada de Pablo, and Colombia (each with populations of less than 5000) in the municipality of Sabanalarga in the Department of Atlántico as control children for a different study of child neurodevelopment (Fig. 1).^29^ All children were born between June 2016 and November 2018 and were evaluated at age 5 or 6 years by local, trained research team members. Eligible children attended school in the cities in which they resided without receiving special education services; did not have chronic illnesses or medical conditions; were born at term following a low-risk pregnancy; did not have any behavioral, neurological, or psychological diagnoses; did not have hearing or vision abnormalities; and did not receive physical, occupational, or speech therapy for a developmental condition based on parent/guardian report.

Children were recruited with informational study flyers shared by classroom teachers in local schools in each city or town. Teachers sent flyers home to parents and passed on contact information of interested families to the study team for eligibility screening and enrollment. A trained research coordinator explained the study and screened parents/guardians for potential exclusion factors; final eligibility determinations were made by the study principal investigator (SM) or site principal investigator (CC).

This study, as well as the other neurodevelopmental outcome study from which data were used,^29^ received approval from the Children’s National Hospital Institutional Review Board, Washington, DC, and the Institutional Review Committee and Independent Committee on Research Ethics (CIRCIE) in Barranquilla, Colombia. Parents or legal guardians provided written informed consent for their child’s participation.

### Neurodevelopmental evaluations

Study visits in Bogotá and Barranquilla took place in groups of 5–10 children in school classroom spaces after the school day had ended. Children from the rural/semi-rural towns in Sabanalarga completed their evaluations at a rented community center space, also in a group setting.^30^ Neuropsychologists and study team members from the same communities administered the child assessments and were trained and overseen by neuropsychologists with experience using the WPPSI and NIH Toolbox Cognition Battery in both clinical practice and research settings (PR, MMB). Children completed ten subtests from the Spanish version of the Wechsler Preschool and Primary Scale of Intelligence-Fourth Edition (WPPSI-IV).^31^ Two selected instruments from the NIH Toolbox Cognition Battery—the Flanker Inhibitory Control and Attention Test and the Pattern Comparison Processing Speed Test—were administered on an iPad.^32^ Parents/caregivers of participating children completed the Behavior Rating Inventory of Executive Function-Second Edition (BRIEF-2) and the Child Behavior Checklist (CBCL) questionnaires to evaluate child executive function and social-emotional development.^33,34^ All questionnaires were scored centrally by research coordinators at Children’s National Hospital.

### Assessment of home and community environments

**Fig. 2:**
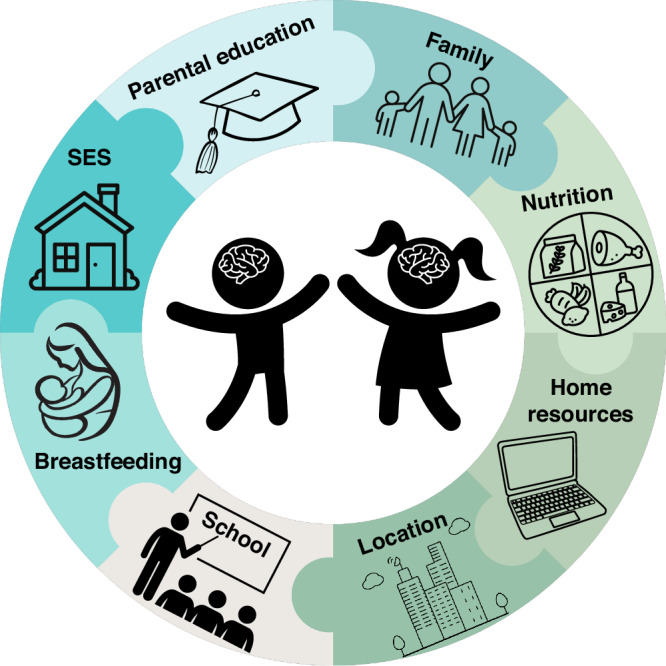
A holistic view of child neurodevelopment, including the myriad of factors that influence cognitive and social-emotional outcomes.

Parents/caregivers completed surveys about their socioeconomic status, family activities and home resources, and maternal and child nutrition (Fig. 2). The socioeconomic questionnaire included questions about the home type and family size, parent/guardian education, occupation, and employment status, childcare, family transportation, and home safety. Parents’ level of education, ranging from none, primary school, secondary school, technical college, or university, was reported. If primary or secondary school was reported as the highest level of education, we asked which was the highest grade level completed, and if they reported technical college or university, we asked for the title of the degree earned.

The family activity survey was an adaptation of the Caldwell HOME inventory.^35^ We modeled our survey after HOME-21 for the twenty-first century^36^ and included questions designed to assess both physical and time-based resources within the household. Physical resources included electronics, musical instruments, and educational materials. In contrast, time-based resources focused on the frequency of caregiver engagement with the child, such as reading, prayer, singing, or play. The adapted HOME inventory excluded a question about hours dedicated to schooling, so the overall score (Total Home Score) was based on 18 items rather than the original 19. In addition to calculating the total HOME inventory score, we also scored physical resources (Physical Home Score, measured on a scale of 0–14) and time-based resources (Time-based Home Score; scale of 0–4) separately.

The maternal and child nutrition questionnaire was an adaptation of the Food Consumption Score questionnaire and inquired about the consumption of dietary groups, including cereals, pulses, vegetables, fruits, meats, dairy products, fats, and sugars.^37^ Responses were structured in a time-range format of 1 week. Caregivers were asked to report how often each food group was consumed during that time period, and whether maternal food consumption patterns at the time of survey completion were similar to their patterns during pregnancy. For meats, the score was applied based on the maximum consumption of either meat, seafood, or eggs, and for sugars, the maximum value between sugars and non-natural beverages was used. Possible scores ranged from 16 to 64, with lower scores indicating poorer/less adequate food consumption. From these questionnaires, we extrapolated Dietary Diversity Scores for both mother and child based on the Maternal Minimum Dietary Diversity questionnaire.^38^ For dietary diversity, a separate score was calculated based on the number of different food categories consumed over the course of 1 week. We excluded beverages and “packaged foods”. Each food category was assigned a score of 1 point if it was consumed on 3 or more days, and 0 points if it was consumed on 0–2 days, for a total score ranging from 0 to 10, with higher scores indicating greater dietary diversity. Additional questions in the nutrition survey collected information about the duration of breastfeeding, formula use, and the introduction of solid foods in infancy.

Covariates of interest were defined and included the number of children in the household, levels of parental education, Total Home Score, Time-based Home Score, Physical Home Score, breastfeeding status, as well as Dietary Diversity Scores and Food Consumption Scores for mothers and children.

### Statistical analysis

Raw data were transferred from the three Colombian sites to Children’s National Hospital via secure Dropbox portal and were entered into REDCap.^39^ Demographic and socioeconomic characteristics of participants were reported as mean (SD) for continuous variables and *N* (percentage) for categorical variables. Neurodevelopmental outcome measures from participants were compared among the three geographic sites. BRIEF T-scores were analyzed for the Behavioral, Emotional, and Cognitive Regulation Indices.^33^ CBCL T-scores for Internalizing Problems and Externalizing Problems were compared among groups.^34^ WPPSI Verbal Comprehension (VCI), Visuospatial (VSI), Fluid Reasoning (FRI), Working Memory (WMI), and Processing Speed Indices (PSI), as well as Full-Scale Intelligence Quotient (FSIQ), were compared among groups. Normative data from Spain were used to calculate WPPSI scores as per the Spanish WPPSI manual.^40^ Age-corrected standard scores generated by the Spanish version of the NIH Toolbox items were used for comparison.^32^

All analyses were conducted using STATA V18 (College Station, TX). A descriptive analysis was conducted to characterize the distribution of the previously discussed outcomes and covariates. The Shapiro-Wilk normality test was performed within each location to assess the normality of each outcome variable and covariate. To assess whether there were differences in mean primary and secondary outcomes between cities, one-way ANOVAs were performed. We defined our primary outcome as WPPSI Full-Scale IQ (FSIQ) and all other outcomes as secondary. If justified, post-hoc pairwise comparisons were conducted, and the Sidak method was used for adjustment for multiple comparisons.

To investigate the impact of different covariates on the primary outcome (WPPSI FSIQ), Spearman rank-order correlations were employed independent of location. This non-parametric method was chosen due to the non-normal distribution and continuous nature of the majority of covariates. This analysis allowed us to identify those covariates that were strongly related to the primary outcome and those that were strongly related to each other to determine which covariates would be included in the final regression model. When two covariates were significantly correlated with one another (*p* < 0.005) and both were significantly correlated with the outcome (*p* < 0.005), only one was chosen for inclusion into the model.

A final multi-variable linear regression model was constructed with WPPSI FSIQ as the dependent variable and location as the independent predictor. This model included meaningful covariates identified during the analysis, specifically total home score, maternal dietary diversity, and maternal education level. Similar models were then also constructed for each secondary outcome. Time and physical resource scores were excluded from the model due to their high correlation with the total home score. Similarly, paternal education and child dietary diversity scores were omitted due to their strong correlation with maternal scores, decreasing the likelihood of model overfitting.

Statistical significance was considered at a *p* value of ≤0.05. Due to the high degree of dependence among the primary and secondary outcomes, the resulting *p* values were not adjusted for multiple testing overall.

## Results

Table 1 summarizes the descriptive statistics and demographic data from Bogotá, Barranquilla, and Sabanalarga cohorts. Observationally, children in Bogotá and Barranquilla had higher mean levels of maternal and paternal education, as well as nutrition, maternal dietary diversity, food consumption scores, and breastfeeding rates compared to children in Sabanalarga (Table 1). Total Home Scores and Physical Home Scores were also higher, observationally, in children from the urban areas, Bogotá and Barranquilla, while the mean number of children in the household was higher in Sabanalarga (Table 1).

**Table 1:** Descriptive characteristics of covariates by location.

Covariate	Barranquilla (*N* = 24)	Bogotá (*N* = 26)	Sabanalarga (*N* = 55)
	Mean ± SD	Mean ± SD	Mean ± SD
Mean age at visit (years)	5.6	5.8	5.2
Range of ages at visit (years)	4.9–6.3	5–6.6	5–5.7
Male sex at birth	11	16	25
Number of children in the household	2.0 ± 0.9	1.5 ± 0.8	2.3 ± 1.5
Maternal education^a^	3.5 ± 0.7	3.4 ± 0.9	2.2 ± 0.7
None	0 (0%)	0 (0%)	1 (1.8%)
Elementary school	0 (0%)	0 (0%)	6 (10.9%)
High school	2 (8.3%)	6 (23.1%)	32 (58.1%)
Technical	9 (37.5%)	4 (15.4%)	15 (27.3%)
University	13 (54.2%)	16 (61.5%)	1 (1.8%)
Paternal education^a,^^b^	3.3 ± 0.8	3.2 ± 0.9	2.0 ± 0.8
None	0 (0%)	0 (0%)	3 (5.5%)
Elementary school	0 (0%)	0 (0%)	2 (3.6%)
High school	5 (20.8%)	7 (26.9%)	37 (67.3%)
Technical	8 (33.3%)	6 (23.1%)	6 (10.9%)
University	11 (45.8%)	11 (42.3%)	2 (3.6%)
Maternal dietary diversity	6.5 ± 2.0	5.8 ± 1.9	3.8 ± 3.1
Maternal food consumption score^c^	50.2 ± 7.7	49.2 ± 4.8	45.8 ± 8.2
Child dietary diversity	6.7 ± 2.0	6.1 ± 1.9	381 ± 3.1
Child food consumption score^d^	49.9 ± 7.6	50.3 ± 5.5	46.5 ± 7.8
Total home score	11.3 ± 2.9	11.7 ± 3.3	7.4 ± 2.7
Time home score	2.0 ± 1.2	1.9 ± 1.2	2.3 ± 1.1
Physical home score	9.3 ± 2.1	9.7 ± 2.7	5.1 ± 2.2
Breastfeeding over a year^e^			
Yes	9 (37.5%)	12 (46.2%)	19 (34.6%)
No	15 (62.5%)	14 (53.9%)	36 (65.4%)
Exclusive breastfeeding^e^			
Yes	8 (33.3%)	14 (53.9%)	19 (34.6%)
No	16 (66.7%)	12 (46.2%)	36 (65.5%)

*SD* standard deviation.

^a^Mean (SD) parental education based on categorical scale: 0 = no formal education, 1 = primary school, 2 = secondary school, 3 = technical college/institute, 4 = university.

^b^Two participants missing in Bogotá cohort, 5 participants missing in Sabanalarga cohort.

^c^Two participants missing in Barranquilla cohort, two missing in Bogotá cohort, 13 missing in Sabanalarga cohort.

^d^Two participants missing in Barranquilla cohort, one missing in Bogotá cohort, 14 missing in Sabanalarga cohort.

^e^Both breastfeeding covariates were yes/no responses; summary statistics are presented as frequency and percent.

Table 2 presents the unadjusted comparisons of neurodevelopmental outcomes across the three locations. Intellectual functioning based on WPPSI FSIQ was below average in Sabanalarga, while mean scores fell in the average range in both Bogotá and Barranquilla.^40^ Mean WPPSI FSIQ in Sabanalarga was significantly lower than in Bogotá and Barranquilla (*p* < 0.001). These group differences were consistent across both verbal (VCI) and nonverbal (VSI; FRI) reasoning scores, as well as working memory and processing speed (WMI; PSI); children in Sabanalarga scored lower across all comparisons. On the NIH toolbox items, children in Sabanalarga scored significantly lower than the Barranquilla cohort on the Flanker test, but we observed no differences on the Pattern Comparison test.

**Table 2:** Descriptive characteristics of primary and secondary outcomes by location.

Outcome	Barranquilla (*N* = 24)	Bogotá (*N* = 26)	Sabanalarga (*N* = 55)	*P* value
	Mean ± SD	Mean ± SD	Mean ± SD	
CBCL T-Scores				
Internalizing Problems	51.7 ± 8.3	57.0 ± 7.9	54.7 ± 9.8	0.13
Externalizing Problems	46.9 ± 7.5^a^	56.5 ± 9.9^a,b^	48.8 ± 8.9^b^	**<0.001**
BRIEF T-Scores				
Behavioral Regulation Index	52.5 ± 9.4	55.0 ± 12.2	52.2 ± 8.7	0.48
Emotional Regulation Index	49.6 ± 5.6	52.2 ± 11.6	51.8 ± 7.8	0.45
Cognitive Regulation Index	47.7 ± 6.6^a^	55.1 ± 10.7^a,b^	48.5 ± 6.7^b^	**<0.001**
WPPSI-IV Standard Scores				
Verbal Comprehension Index	94.9 ± 17.7^a^	99.5 ± 13.8^b^	76.7 ± 17.0^a,b^	**<0.001**
Visual Spatial Index	95.8 ± 12.1^a^	99.5 ± 13.7^b^	79.0 ± 11.6^a,b^	**<0.001**
Fluid Reasoning Index	91.5 ± 14.1^a,b^	106.3 ± 13.3^a,c^	75.1 ± 11.7^b,c^	**<0.001**
Working Memory Index	95.9 ± 10.5^a^	101.3 ± 13.5^b^	83.2 ± 11.7^a,b^	**<0.001**
Processing Speed Index	106.2 ± 13.6^a,b^	96.7 ± 95.5^a,c^	88.3 ± 13.3^b,c^	**<0.001**
Full-Scale IQ	96.7 ± 14.1^a^	100.9 ± 11.2^b^	76.0 ± 13.6^a,b^	**<0.001**
NIH Toolbox Standard Scores				
Pattern Comparison Test^d^	84.9 ± 17.5	85.3 ± 11.2	76.5 ± 14.1	0.11
Flanker Test^e^	105.8 ± 11.5^a^	103.1 ± 10.6	93.2 ± 21.1^a^	**0.005**

*SD* standard deviation, *CBCL* Child Behavior Checklist, *BRIEF* Behavior Rating Inventory of Executive Function, *WPPSI* Wechsler Preschool and Primary Scale of Intelligence, *IQ* intelligence quotient.

^a,b,c^*p* values less than 0.05 are bolded. For each outcome, a shared subscript between groups indicates statistically significantly different means after adjustment for multiple comparisons.

^d^Due to the removal of values of 54 (equivalent to a score of 0, likely due to a technological system error), the number of responses is *N* = 19 for Barranquilla, *N* = 16 for Bogotá, and *N* = 22 for Sabanalarga.

^e^Due to a missing value and the removal of a value of 54 (equivalent to a score of 0, likely due to a technological system error), the number of responses is *N* = 24 for Bogotá.

Average parent ratings of executive functioning were in the normal range across all groups; however, the Cognitive Regulation Index (CRI) and externalizing problems on the CBCL were more elevated for children from Bogotá than those from Barranquilla and Sabanalarga (*p* < 0.001). There were no significant differences among groups for behavioral regulation (BRI), emotional regulation (ERI), or internalizing problems.

### Final multi-variable models adjusting for covariates

#### WPPSI-IV

Table 3 presents the final model for WPPSI FSIQ score; the differences between Sabanalarga and both Bogotá (*p* < 0.001) and Barranquilla (*p* = 0.018) remained even after adjusting for the covariates. This model explained 46% of the variance (*R*^2^ = 0.4583), with maternal education being the most significant factor (*p* < 0.001).

**Table 3:** The effects of social and environmental factors on WPPSI Full-Scale IQ score.

Model term	Coefficient	*p* value	Location	Adjusted mean ± SE	
Location	−4.97	0.019	Barranquilla	91.69 ± 2.90	Bogotá vs. Barranquilla (*p* = 0.47)
Total home score	0.75	0.10	Bogotá	96.30 ± 2.76	Sabanalarga vs. Barranquilla (*p* = 0.018)
Dietary diversity—mom	0.42	0.41	Sabanalarga	80.41 ± 2.07	Sabanalarga vs. Bogotá (***p* < 0.001**)
Maternal education	6.64	<0.001			

Model *R*^2^ = 0.4583.

Left: Multi-variable model assessing WPPSI FSIQ score and significant, non-interrelated covariates of location, total home resources, maternal dietary diversity during pregnancy, and maternal education. Right: Mean WPPSI FSIQ adjusted by location and comparisons between locations. Covariates in adjusted comparisons were set to the mean for combined locations.

*p* values less than 0.05 are bolded.

*WPPSI* Wechsler Preschool and Primary Scale of Intelligence, *SE* standard error.

Supplementary tables are provided for the final model representing the subscales or index scores from measures. Differences among groups were evident for all the WPPSI index scores (Tables S1–S5). Specifically, for the WPPSI VSI, scores from Sabanalarga were significantly lower than both Bogotá and Barranquilla before adjustment. However, after adjustment, only the difference between Sabanalarga and Bogotá remained significant (*p* = 0.008, Table S1). Similarly, for WPPSI FRI, differences among all three locations were found before adjustment, but after adjusting for covariates, the differences persisted between Bogotá and the other locations, but the difference between Sabanalarga and Barranquilla was no longer significant (Table S2). The WPPSI WMI showed persistent differences between Sabanalarga and both Bogotá and Barranquilla after adjustment (Table S3). For the WPPSI PSI, differences persisted between Sabanalarga and Barranquilla after adjustment (*p* = 0.022), but the difference between Sabanalarga and Bogotá disappeared (Table S4). Finally, the difference in WPPSI VCI was significant before adjustment but did not remain significant after adjusting for covariates (Table S5).

#### CBCL

After adjusting for covariates, behavioral outcomes had fewer significant differences. For Externalizing problems, differences between Bogotá and Barranquilla remained significant after adjustment (*p* = 0.001, Table S6), but no difference was observed between Bogotá and Sabanalarga. For Internalizing problems, no differences were found across locations (Table S7).

#### NIH Toolbox

For NIH Toolbox outcomes, including the Pattern Comparison Processing Speed test and Flanker Inhibitory Control and Attention Test, there were no differences among locations after adjustment (Tables S8 and S9). All scores on the Pattern Comparison Processing Speed test were below average across locations, while Flanker test scores remained within normal range.

#### BRIEF-2

In the BRIEF, adjusted CRI scores were elevated in children from Bogotá compared to Sabanalarga (*p* = 0.03; Table S10). However, there were no differences in Emotional Regulation Index (ERI) or Behavioral Regulation Index (BRI) scores after adjustment, and all scores remained within the normal range (Tables S11 and S12).

#### Explained variability (*R*^2^)

The *R*^2^ values for the final models, presented in the supplementary tables, varied across outcomes (Tables S1–S12). Cognitive measures from the WPPSI, including the FSIQ (*R*^2^ = 0.4583, Table 3) and Fluid Reasoning Index (*R*^2^ = 0.4542, Table S2) were better explained by the models, while behavioral outcomes like CBCL Internalizing problems (*R*^2^ = 0.0209, Table S7) and BRIEF CRI (*R*^2^ = 0.0248, Table S10) had lower explanatory power.

## Discussion

The study found differences in cognitive, executive functioning, and social-emotional outcomes among Colombian children living in different regions, influenced by maternal education, dietary diversity, and home resources. We utilized a multi-domain neurodevelopmental outcome toolbox in a total of 105 children aged 5–6 years old from Bogotá, Barranquilla, and Sabanalarga, all of whom are considered typically developing in their communities. To our knowledge, this is the first study to compare school-age developmental outcomes in healthy children across multiple regions of Colombia, a large and heterogeneous country in Latin America. However, findings also provide important rationale for directing further attention to children in other countries where geographic disparities exist among school and educational quality, community resources, and home environmental factors. It is also necessary to understand the normative developmental scores for children in their unique contexts when considering whether development is typical or atypical, and when comparing outcomes to those of other cohorts. The United Nations Sustainable Development Goals 2015–2030 emphasize the importance of ensuring healthy development and well-being for all children, with particular attention to those at risk of poor neurodevelopmental outcomes.^41,42^ Proper measurement and interpretation of neurodevelopmental data are particularly important when the goal is to understand the impact of regionally specific events, such as viral exposure. We hope our findings will add to the growing literature on neurodevelopmental outcomes in Latin America and support future efforts to enhance early developmental screening and intervention.

As hypothesized, we found that cognitive functioning, as measured by the WPPSI-IV, was higher in cohorts from the urban cities of Bogotá and Barranquilla compared to the rural municipality of Sabanalarga. We observed this difference across all indices of the WPPSI-IV. Prior studies also show that children in urban areas who often have greater access to educational resources, healthcare, and enriched home environments have enhanced cognitive development compared to children growing up in non-urban areas.^43–45^ These disparities may be even larger in low and middle-income countries of Latin America.^46,47^ For example, research in Brazil by Brito et al. highlighted how rural settlements can be a defining factor for vulnerabilities such as food insecurity and malnutrition, and how their impact in early childhood can significantly affect cognitive development.^46^ Similarly, Hermida et al. identified increased cognitive developmental risks for school-aged children in rural Argentina when compared to urban counterparts, related to lower preschool attendance and lower completed level of education among children’s fathers.^47^ These findings align with our results, indicating that early school-age cognitive outcomes, such as IQ, are highly vulnerable to environmental influences such as access to community resources, parental education, and nutrition.

Across other neurodevelopmental domains, some statistically significant group differences were found; however, participants were functioning within age expectations with no clinically elevated concerns overall. We observed intergroup differences on the Externalizing domain of the CBCL, the Cognitive Regulation domain of the BRIEF-2, and the NIH Toolbox Flanker Inhibitory Control and Attention Test. Although these differences all occurred within average score ranges, it is important to note that the linear regression models for these outcomes indicate that group differences seem to be less influenced by dietary diversity, maternal education, home resources, and location than the cognitive differences observed; thus, adjusting for these covariates did not have a large impact on the group means nor change the statistical conclusions of the comparison. The relatively smaller statistical effect of these covariates in comparison to cognitive outcomes was an unexpected finding, as other studies of early school-age children from across the world have found strong positive correlations between the quality of the home environment, including caregiving practices, nutrition, and environmental stimulation, to positively mediate social-emotional skills.^1,2^ However, a study of Irish children ages 6 months to 3 years found that improvements to their home environment through an intervention program did not account for improvements in child emotional outcomes as measured by the Short Early Development Inventory.^4^ It is possible that at this early age, attendance at school or other external childcare may provide a common ground for social-emotional development that helps to mediate variable home environmental and community factors.^48^

Within our comparison of these cohorts of typically developing children who, as expected, generally performed within age expectations, we found that some outcomes were much more sensitive to environmental factors than others. At maximum, covariates accounted for approximately 50% of the variability observed between groups. Once maternal education, dietary diversity, home resources, and location differences were mathematically equalized, significant differences in measured intellectual function persisted in our primary outcome measure, the WPPSI-IV FSIQ. Standardized tests like the WPPSI are widely used to measure cognitive functioning globally, and IQ is often the primary or sole measure that researchers utilize to describe neurodevelopmental outcomes.^16^ However, such measures may be vulnerable to cultural and environmental differences that affect performance.^16^ Specifically, the normative data for the Spanish WPPSI-IV used in this study were based on children from Spain who, despite sharing a language generally, are different culturally and linguistically from children in Latin America. Notably, we found that the social-emotional and executive function measures were less impacted by external influences than the WPPSI-IV. Thus, while studies should strive to include well-matched control comparison groups to minimize these influences, using measures that are less prone to cultural and environmental biases may also be an effective strategy.

While this study is strengthened by the robust data collected from three distinct populations in Colombia, there are still numerous factors that were not measured within these cohorts. Variables such as time spent in school, caregiver skills, and education (distinct from parental education, as those who actually care for children outside of school hours may not be the child’s parents), disruptions in education and socialization due to the COVID-19 pandemic,^49^ and genetic and neurological data were not examined as a part of this study. These variables may be important to include, given that children in this study attended different types of schools; the Bogotá cohort was enrolled in a private school, while children in Barranquilla and Sabanalarga attended public schools. However, we inherently accounted for this variability by including location as a covariate in our regression model and continued to see differences in neurodevelopmental outcomes even after this adjustment. Furthermore, the primary disparity in outcomes fell along the lines of rural and urban settings (Sabanalarga vs Bogotá/Barranquilla), rather than between children attending a public or private school.

In addition, the overall sample size was small and differed between groups due to the utilization of both data from another developmental study^29^ as well as data collected specifically for this analysis as part of an exploratory pilot award. The small sample size may limit the generalizability of the study to other populations where resources and environmental factors differ. An additional limitation in our interpretation of these results is the fact that the neurodevelopmental assessment measures utilized were not developed for Colombian populations, nor were normative data available from Colombia, although the WPPSI is a commonly used test in Spanish-speaking Latin America at this age.^16^ However, we would expect this to impact the scores of each group relatively equally, and thus the group comparison is valid.

Overall, this study demonstrates the critical role of selecting well-matched control children and adequate outcome measures in a case-control developmental study design. Despite the propensity of many researchers to rely on statistical correction of social and environmental factors such as family socioeconomic status, parental education, nutrition, and home resources, this is an imperfect approach to addressing these powerful mediators of child development. Many unmeasured factors, such as cultural norms, neighborhood safety, community support, and access to healthcare, likely contributed to the observed variability, reflecting the complexity of isolating environmental influences. Similar limitations have been noted in other studies, which highlight how cultural practices and community stressors can shape child outcomes.^50,51^ Furthermore, IQ and other cognitive measures may not always be the preferred means of assessing neurodevelopmental performance, given their vulnerability to early-life experiences and cultural bias. Future work would benefit from further elucidating additional factors that account for variability in neurodevelopmental outcomes. This involves exploring more detailed educational variables like time spent in school and teaching quality, as well as addressing socioeconomic aspects such as food insecurity. A holistic approach that considers broader environmental, genetic, and sociocultural factors is critical to understand the complex nature of neurodevelopment in children.

## Conclusion

Social and environmental influences are critical components of a well-rounded understanding of child neurodevelopment, particularly in populations with large disparities in child opportunity. These factors have been historically understudied in LMICs such as Colombia. In this pilot study, we found that neurodevelopmental outcomes spanning cognitive and social-emotional domains differed between typically developing children from disparate regions of Colombia, and were influenced by maternal education, dietary diversity, and home resources. However, even the numerous covariates examined in this study explained only approximately half of the variability between groups. This study underscores the importance of improved approaches to understanding the complexity of drivers of child development in different populations. Future research in child development should ensure that control populations are well-matched to the population of interest, as statistical correction is not sufficient to fully account for these differences. Standardized assessment measures should be chosen with the population of interest in mind to minimize confounding due to social, cultural, and environmental differences.

## Supplementary information

Supplementary Material

## Data Availability

De-identified data are available from the corresponding author upon reasonable request.
