# Supplementary material for: Accounting for social and environmental drivers of neurodevelopment: lessons from a pilot study in a pediatric Colombian cohort
Source: Pediatr Res. 2025 Nov 4;99(7):2660–8. doi: 10.1038/s41390-025-04452-9 (PMC13135377; doi:10.1038/s41390-025-04452-9)
Supplement: Supplementary file 1 [file 41390_2025_4452_MOESM1_ESM.pdf]

Supplementary Material

Table S1. The effects of social and environmental factors on WPPSI Visual Spatial Index.

**Model term**	**Coefficient**	**p-value**
Location	-2.70	0.15
Total Home Score	1.44	0.001
Maternal Dietary Diversity	0.48	0.29
Maternal Education	4.56	0.006

**Location**	**Adjusted mean± SE**	
Barranquilla	90.29 ± 2.68	Bogota vs. Barranquilla (p=0.59)
Bogota	94.03 ± 2.56	Sabanalarga vs. Barranquilla (p=0.26)
Sabanalarga	84.04 ± 1.91	Sabanalarga vs. Bogota **(p=0.019)**

Model $r^2 = 0.4505$

Left: Multi-variable model assessing WPPSI Visual spatial standard score and significant, non-interrelated covariates of location, total home resources, maternal dietary diversity during pregnancy and maternal education. Right: Mean WPPSI Visual spatial standard score adjusted by location and comparisons between locations. Covariates in adjusted comparisons were set to mean for combined locations.

WPPSI: Wechsler Preschool and Primary Scale of Intelligence; SE: standard error. p-values less than 0.05 are bolded.

Table S2. The effects of social and environmental factors on WPPSI Fluid Reasoning Index.

**Model term**	**Coefficient**	**p-value**
Location	-1.83	0.41
Total Home Score	1.43	0.003
Maternal Dietary Diversity	0.45	0.40
Maternal Education	7.33	<0.001

**Location**	**Adjusted mean± SE**	
Barranquilla	85.88 ± 2.73	Bogota vs. Barranquilla **(p<0.001)**
Bogota	100.91 ± 2.60	Sabanalarga vs. Barranquilla (p=0.33)
Sabanalarga	80.04 ± 1.95	Sabanalarga vs. Bogota **(p<0.001)**

Model $r^2 = 0.4542$

Left: Multi-variable model assessing WPPSI Fluid reasoning standard score and significant, non-interrelated covariates of location, total home resources, maternal dietary diversity during pregnancy and maternal education. Right: Mean WPPSI Fluid reasoning standard score adjusted by location and comparisons between locations. Covariates in adjusted comparisons were set to mean for combined locations.

WPPSI: Wechsler Preschool and Primary Scale of Intelligence; SE: standard error. p-values less than 0.05 are bolded.

Table S3. The effects of social and environmental factors on WPPSI Working Memory Index.

**Model term**	**Coefficient**	**p-value**
Location	-4.87	0.018
Total Home Score	-0.11	0.80
Maternal Dietary Diversity	0.17	0.72
Maternal Education	2.91	0.029

**Location**	**Adjusted mean± SE**	
Barranquilla	94.97 ± 2.77	Bogota vs. Barranquilla (p=0.24)
Bogota	100.80 ± 2.64	Sabanalarga vs. Barranquilla **(p=0.013)**
Sabanalarga	83.82 ± 1.97	Sabanalarga vs. Bogota **(p<0.001)**

Model $r^2 = 0.2598$

Left: Multi-variable model assessing WPPSI Working memory standard score and significant, non-interrelated covariates of location, total home resources, maternal dietary diversity during pregnancy and maternal education. Right: Mean WPPSI Working memory standard score adjusted by location and comparisons between locations. Covariates in adjusted comparisons were set to mean for combined locations.

WPPSI: Wechsler Preschool and Primary Scale of Intelligence; SE: standard error. p-values less than 0.05 are bolded.

Table S4. The effects of social and environmental factors on WPPSI Processing Speed Index.

**Model term**	**Coefficient**	**p-value**

**Location**	**Adjusted mean± SE**	

				Location	Adjusted mean± SE	
Location	-5.81	0.005		Barranquilla	102.54 ± 2.92	Bogota vs. Barranquilla **(p=0.043)**
Total Home Score	0.18	0.67		Bogota	93.65 ± 2.79	Sabanalarga vs. Barranquilla **(p=0.019)**
Maternal Dietary Diversity	0.83	0.09		Sabanalarga	91.33 ± 2.08	Sabanalarga vs. Bogota (p=0.91)
Maternal Education	2.09	0.23				

Model r^2 = 0.2925

Left: Multi-variable model assessing WPPSI Processing speed standard score and significant, non-interrelated covariates of location, total home resources, maternal dietary diversity during pregnancy and maternal education. Right: Mean WPPSI Processing speed standard score adjusted by location and comparisons between locations. Covariates in adjusted comparisons were set to mean for combined locations.

WPPSI: Wechsler Preschool and Primary Scale of Intelligence; SE: standard error. p-values less than 0.05 are bolded.

Table S5. The effects of social and environmental factors on WPPSI Verbal Comprehension Index.

**Model term**	**Coefficient**	**p-value**		**Location**	**Adjusted mean± SE**	
Location	-2.51	0.34		Barranquilla	89.92 ± 3.72	Bogota vs. Barranquilla (p=0.60)
Total Home Score	0.62	0.27		Bogota	95.05 ± 3.55	Sabanalarga vs. Barranquilla (p=0.57)
Maternal Dietary Diversity	0.49	0.44		Sabanalarga	83.92 ± 2.65	Sabanalarga vs. Bogota (p=0.08)
Maternal Education	6.21	0.007				

Model r^2 = 0.2534

Left: Multi-variable model assessing WPPSI Verbal comprehension standard score and significant, non-interrelated covariates of location, total home resources, maternal dietary diversity during pregnancy and maternal education. Right: Mean WPPSI Verbal comprehension standard score adjusted by location and comparisons between locations. Covariates in adjusted comparisons were set to mean for combined locations.

WPPSI: Wechsler Preschool and Primary Scale of Intelligence; SE: standard error.

Table S6. The effects of social and environmental factors on CBCL Externalizing Problems T-score.

**Model term**	**Coefficient**	**p-value**		**Location**	**Adjusted mean± SE**	
Location	2.24	0.14		Barranquilla	46.01 ± 2.06	Bogota vs. Barranquilla **(p=0.001)**
Total Home Score	0.17	0.59		Bogota	55.74 ± 1.96	Sabanalarga vs. Barranquilla (p=0.52)
Maternal Dietary Diversity	0.05	0.88		Sabanalarga	49.52 ± 1.47	Sabanalarga vs. Bogota (p=0.08)
Maternal Education	2.56	0.051				

Model r^2 = 0.0563

Left: Multi-variable model assessing CBCL Externalizing problems T-score and significant, non-interrelated covariates of location, total home resources, maternal dietary diversity during pregnancy and maternal education. Right: Mean CBCL Externalizing problems T-score adjusted by location and comparisons between locations. Covariates in adjusted comparisons were set to mean for combined locations.

CBCL: Child Behavior Checklist; SE: standard error. p-values less than 0.05 are bolded.

Table S7. The effects of social and environmental factors on CBCL Internalizing Problems T-score.

**Model term**	**Coefficient**	**p-value**		**Location**	**Adjusted mean± SE**	
Location	1.94	0.19		Barranquilla	51.45 ± 2.13	Bogota vs. Barranquilla (p=0.13)
Total Home Score	-0.04	0.91		Bogota	56.81 ± 2.03	Sabanalarga vs. Barranquilla (p=0.57)
Maternal Dietary Diversity	-0.004	0.99		Sabanalarga	54.88 ± 1.52	Sabanalarga vs. Bogota (p=0.87)
Maternal Education	1.33	0.30				

Model r^2 = 0.0209
Left: Multi-variable model assessing CBCL Internalizing problems T-score and significant, non-interrelated covariates of location, total home resources, maternal dietary diversity during pregnancy and maternal education. Right: Mean CBCL Internalizing problems T-score adjusted by location and comparisons between locations. Covariates in adjusted comparisons were set to mean for combined locations.
CBCL: Child Behavior Checklist; SE: standard error.

Table S8. The effects of social and environmental factors on NIH Toolbox Pattern Comparison Test.

**Model term**	**Coefficient**	**p-value**
Location	-1.95	0.50
Total Home Score	1.09	0.01
Maternal Dietary Diversity	0.23	0.82
Maternal Education	0.31	0.92

**Location**	**Adjusted mean± SE**	
Barranquilla	82.63 ± 4.38	Bogota vs. Barranquilla (p=0.99)
Bogota	81.87 ± 4.78	Sabanalarga vs. Barranquilla (p=0.85)
Sabanalarga	78.27 ± 3.41	Sabanalarga vs. Bogota (p=0.93)

Model r^2 = 0.1168
Left: Multi-variable model assessing NIH Toolbox PC Test score and significant, non-interrelated covariates of location, total home resources, maternal dietary diversity during pregnancy and maternal education. Right: Mean NIH Toolbox PC Test score adjusted by location and comparisons between locations. Covariates in adjusted comparisons were set to mean for combined locations.
PC: Pattern Comparison; SE: standard error.

Table S9. The effects of social and environmental factors on NIH Toolbox Flanker Test.

**Model term**	**Coefficient**	**p-value**
Location	-2.09	0.44
Total Home Score	0.18	0.76
Maternal Dietary Diversity	0.21	0.76
Maternal Education	5.52	0.023

**Location**	**Adjusted mean± SE**	
Barranquilla	101.22 ± 3.93	Bogota vs. Barranquilla (p=0.95)
Bogota	98.82 ± 3.91	Sabanalarga vs. Barranquilla (p=0.83)
Sabanalarga	97.09 ± 2.80	Sabanalarga vs. Bogota (p=0.98)

Model r^2 = 0.1605
Left: Multi-variable model assessing NIH Toolbox Flanker Test score and significant, non-interrelated covariates of location, total home resources, maternal dietary diversity during pregnancy and maternal education. Right: Mean NIH Toolbox Flanker score adjusted by location and comparisons between locations. Covariates in adjusted comparisons were set to mean for combined locations.
SE: standard error.

Table S10. The effects of social and environmental factors on BRIEF CRI T-score.

**Model term**	**Coefficient**	**p-value**
Location	0.93	0.49
Total Home Score	-0.002	0.99
Maternal Dietary Diversity	0.15	0.63
Maternal Education	1.47	0.21

**Location**	**Adjusted mean± SE**	
Barranquilla	47.55 ± 1.84	Bogota vs. Barranquilla **(p=0.003)**
Bogota	55.17 ± 1.76	Sabanalarga vs. Barranquilla (p=0.97)
Sabanalarga	48.55 ± 1.31	Sabanalarga vs. Bogota **(p=0.025)**

Model r^2 = 0.0248
Left: Multi-variable model assessing BRIEF CRI T-score and significant, non-interrelated covariates of location, total home resources, maternal dietary diversity during pregnancy and maternal education. Right: Mean BRIEF CRI T-score adjusted by location and comparisons between locations. Covariates in adjusted comparisons were set to mean for combined locations.

BRIEF BRI: Behavior Rating Inventory of Executive Function Cognitive Regulation Index; SE: standard error. p-values less than 0.05 are bolded.

Table S11. The effects of social and environmental factors on BRIEF BRI T-score.

**Model term**	**Coefficient**	**p-value**
Location	1.42	0.36
Total Home Score	0.09	0.78
Maternal Dietary Diversity	-0.07	0.86
Maternal Education	1.47	0.07

**Location**	**Adjusted mean± SE**	
Barranquilla	50.92 ± 2.28	Bogota vs. Barranquilla (p=0.73)
Bogota	53.51 ± 2.18	Sabanalarga vs. Barranquilla (p=0.78)
Sabanalarga	53.61 ± 1.63	Sabanalarga vs. Bogota (p=0.99)

Model $r^2 = 0.0421$

Left: Multi-variable model assessing BRIEF BRI T-score and significant, non-interrelated covariates of location, total home resources, maternal dietary diversity during pregnancy and maternal education. Right: Mean BRIEF BRI T-score adjusted by location and comparisons between locations. Covariates in adjusted comparisons were set to mean for combined locations.

BRIEF BRI: Behavior Rating Inventory of Executive Function Behavior Regulation Index; SE: standard error.

Table S12. The effects of social and environmental factors on BRIEF ERI T-score.

**Model term**	**Coefficient**	**p-value**
Location	2.03	0.14
Total Home Score	-0.05	0.87
Maternal Dietary Diversity	-0.07	0.84
Maternal Education	1.73	0.15

**Location**	**Adjusted mean± SE**	
Barranquilla	48.58 ± 1.99	Bogota vs. Barranquilla (p=0.58)
Bogota	51.38 ± 1.90	Sabanalarga vs. Barranquilla (p=0.39)
Sabanalarga	52.55 ± 1.41	Sabanalarga vs. Bogota (p=0.96)

Model $r^2 = 0.0314$

Left: Multi-variable model assessing BRIEF ERI T-score. and significant, non-interrelated covariates of location, total home resources, maternal dietary diversity during pregnancy and maternal education. Right: Mean BRIEF ERI T-score. adjusted by location and comparisons between locations. Covariates in adjusted comparisons were set to mean for combined locations.

BRIEF ERI: Behavior Rating Inventory of Executive Function Emotional Regulation Index; SE: standard error.
